# Self-Masked Aldehyde Inhibitors of Human Cathepsin L Are Potent Anti-CoV-2 Agents

**DOI:** 10.3389/fchem.2022.867928

**Published:** 2022-07-04

**Authors:** Jiyun Zhu, Linfeng Li, Aleksandra Drelich, Bala C. Chenna, Drake M. Mellott, Zane W. Taylor, Vivian Tat, Christopher Z. Garcia, Ardala Katzfuss, Chien-Te K. Tseng, Thomas D. Meek

**Affiliations:** ^1^ Department of Biochemistry and Biophysics, College of Agriculture and Life Sciences, Texas A&M University College Station, College Station, TX, United States; ^2^ Department of Microbiology and Immunology, John Sealy School of Medicine, University of Texas Medical Branch at Galveston, Galveston, TX, United States; ^3^ Department of Chemistry, College of Science, Texas A&M University College Station, College Station, TX, United States

**Keywords:** SARS coronavirus-2, cathepsin L, self-masked aldehydes, reversible covalent inactivation, COVID-19, cysteine proteases

## Abstract

Cysteine proteases comprise an important class of drug targets, especially for infectious diseases such as Chagas disease (cruzain) and COVID-19 (3CL protease, cathepsin L). Peptide aldehydes have proven to be potent inhibitors for all of these proteases. However, the intrinsic, high electrophilicity of the aldehyde group is associated with safety concerns and metabolic instability, limiting the use of aldehyde inhibitors as drugs. We have developed a novel class of compounds, self-masked aldehyde inhibitors (SMAIs) which are based on the dipeptide aldehyde inhibitor (Cbz-Phe-Phe-CHO, **1**), for which the P_1_ Phe group contains a 1′-hydroxy group, effectively, an *o*-tyrosinyl aldehyde (Cbz-Phe-*o*-Tyr-CHO, **2**; (Li *et al.* (2021) *J. Med. Chem. 64,* 11,267–11,287)). Compound **2** and other SMAIs exist in aqueous mixtures as stable δ-lactols, and apparent catalysis by the cysteine protease cruzain, the major cysteine protease of *Trypanosoma cruzi*, results in the opening of the lactol ring to afford the aldehydes which then form reversible thiohemiacetals with the enzyme. These SMAIs are also potent, time-dependent inhibitors of human cathepsin L (*K*_i_ = 11–60 nM), an enzyme which shares 36% amino acid identity with cruzain. As inactivators of cathepsin L have recently been shown to be potent anti-SARS-CoV-2 agents in infected mammalian cells (Mellott *et al.* (2021) *ACS Chem. Biol. 16*, 642–650), we evaluated SMAIs in VeroE6 and A549/ACE2 cells infected with SARS-CoV-2. These SMAIs demonstrated potent anti-SARS-CoV-2 activity with values of EC_50_ = 2–8 μM. We also synthesized pro-drug forms of the SMAIs in which the hydroxyl groups of the lactols were O-acylated. Such pro-drug SMAIs resulted in significantly enhanced anti-SARS-CoV-2 activity (EC_50_ = 0.3–0.6 μM), demonstrating that the O-acylated-SMAIs afforded a level of stability within infected cells, and are likely converted to SMAIs by the action of cellular esterases. Lastly, we prepared and characterized an SMAI in which the sidechain adjacent to the terminal aldehyde is a 2-pyridonyl-alanine group, a mimic of both phenylalanine and glutamine. This compound (**9**) inhibited both cathepsin L and 3CL protease at low nanomolar concentrations, and also exerted anti-CoV-2 activity in an infected human cell line.

## Introduction

The global COVID-19 pandemic, caused by the β-coronavirus SARS-CoV-2, has, as of the end of 2021, resulted in 289 million cases and 5.4 million deaths worldwide (Johns Hopkins University of Medicine, 2022). The socio–economic consequences of this pandemic are difficult to overstate. Despite the availability of effective vaccines since 2021, and the recent (emergency use) approval of a bespoke, small-molecule drug (Paxlovid (Pfizer, 2021); nirmatrelvir, combined with ritonavir) for the treatment of COVID-19, the emergence of new variant forms of the SARS-CoV-2 virus, such as the delta and omicron strains, will require further discovery of new therapeutic agents to counter the inevitable development of SARS-CoV-2 variants that are resistant to these first-generation drugs.

**SCHEME 1:**
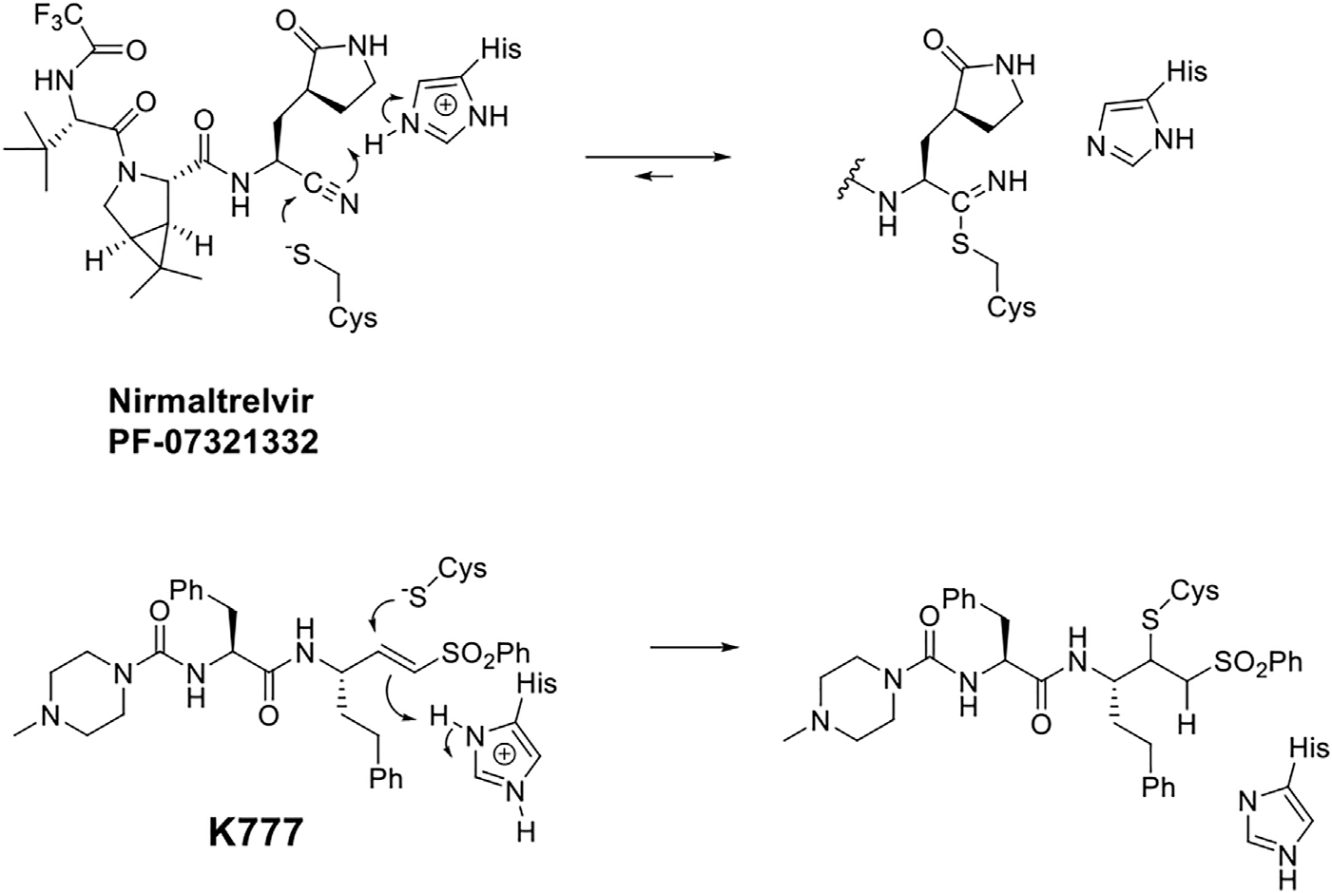
Putative Chemical Mechanisms of Reversible Covalent Inhibition of SARS CoV-2 3CL Protease by Nirmaltrelvir (top) and Irreversible Covalent Inactivation of Human Cathepsin L by **K777** (bottom).

The virally-encoded cysteine protease known as the Main protease, or 3CL protease (Zhang et al., 2020; Dai et al., 20202020; and Mellott et al., 2021), is the target of nirmatrelvir, also known as **PF-07321332** (Owen et al., 2021) (Scheme 1). **PF-07321332** exerts reversible covalent inhibition of the 3CL protease by a putative mechanism of action involving the formation of a thioimidate adduct (Scheme 1) (Bai et al., 2021; Owen et al., 2021).

In general, the most effective inhibitors of cysteine proteases are peptide analogues bearing electrophilic warheads, which undergo reaction with the active-site cysteine (Puzer et al., 2004; Cianni et al., 2019; and Cianni et al., 2021). The peptide or peptidomimetic moieties of such inhibitors are based on the substrate specificity of the target cysteine protease, and are therefore designed to selectively guide the appended warhead to the active-site of intent.

In this regard, the preferred peptide substrates of the cysteine protease cathepsin L (Brömme et al., 1994; Puzer et al., 2004) and cruzain (Zhai and Meek, 2018) contain leucine, phenylalanine, or a similar amino-acid sidechain at the P_2_ (Schechter and Berger, 1967) of the peptide, while for the CoV-2 3CL protease, a virtually invariant glutamine is found at the P_1_ position, and leucine and other hydrophobic amino-acid sidechains exist at the P_2_ positions (Rut et al., 2021). These preferences inform the development of specific peptide scaffolds for the inhibitors of these proteases. It is generally held that reversible covalent inhibition will afford inhibitors of higher selectivity and lower cytotoxicity than their irreversible counterparts, such as the vinyl sulfone warhead of K777 (Doyle et al., 2007; Kerr et al., 2009). Peptide aldehydes comprise a long-standing class of reversible covalent inhibitors of the cysteine protease (Lewis and Wolfenden, 1977; Woo et al., 1995; Pehere et al., 2019).

In addition to the essentiality of the 3CL protease to establish cellular infection by SARS-CoV-2, host-cell proteases are also involved, catalyzing the peptidolysis of the coronaviral spike protein (S), which is required for cellular uptake and intracellular trafficking of the coronavirus (Mellott et al., 2021; Hoffmann et al., 2020). Accordingly, these host proteases may also provide additional drug targets for SARS-CoV-2 infection. We recently reported that **K777**, or **K11777**, a di-peptide vinyl sulfone inactivator of the trypanosomal cysteine protease, cruzain (Scheme 1), which had been a clinical candidate for Chagas disease (McKerrow et al., 2009), was an exceptionally potent anti-SARS-CoV-2 agent (Mellott et al., 2021). We used a propargyl analogue of **K777** to covalently label and characterize the cellular target of **K777** in SARS-CoV-2-infected Vero E6 cells. We showed that cathepsin L was specifically labeled by **K777**, and in addition, we demonstrated that purified human cathepsin L catalyzed a unique cleavage of the spike protein from SARS-CoV-2; an event that assists the coronaviral uptake. As a result, human cathepsin L comprises an additional cellular target for the development of anti–COVID-19 agents.

**FIGURE 1:**
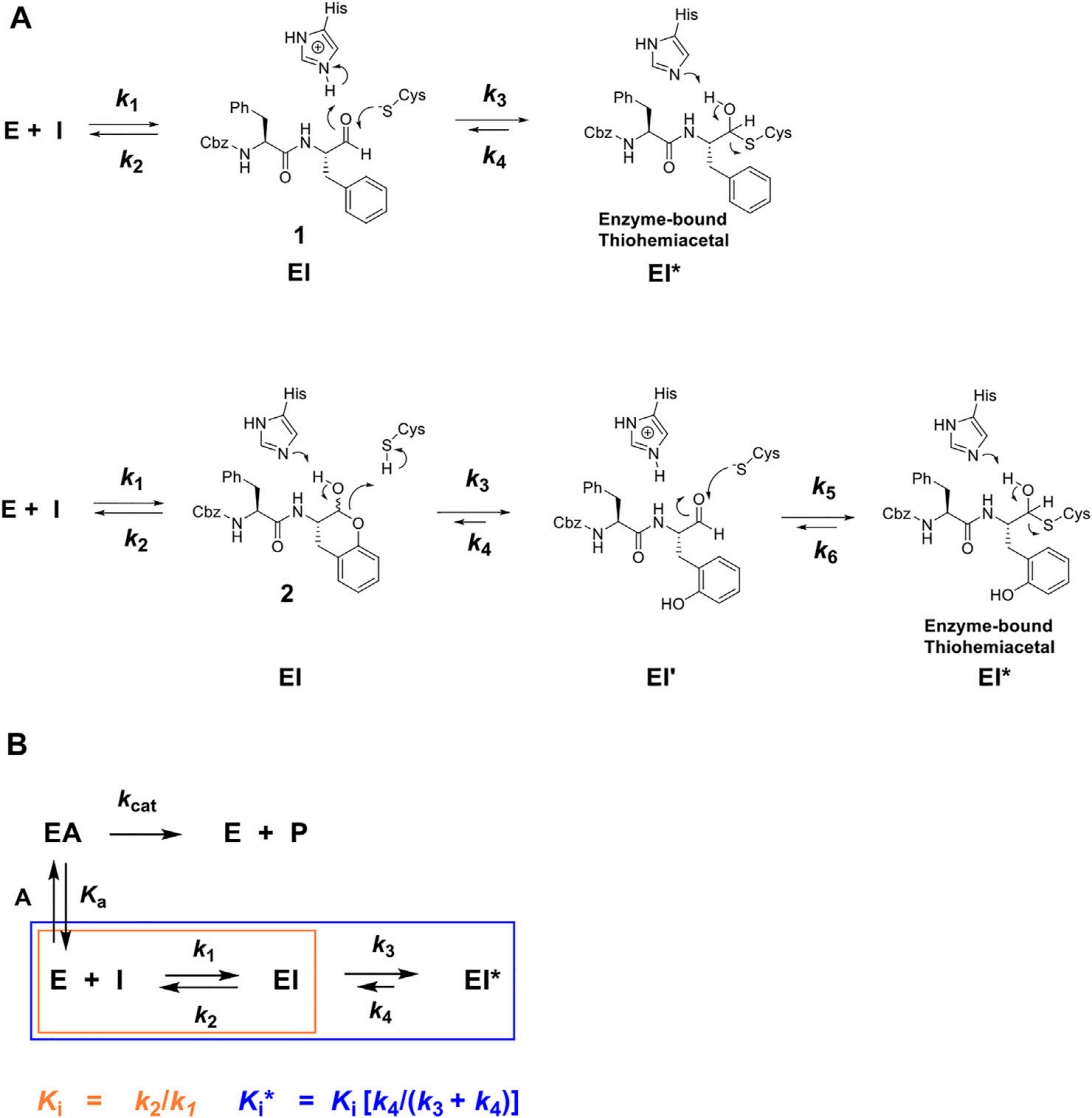
**(A)** Putative chemical mechanisms of reversible covalent inhibition of a cysteine protease by Cbz-Phe-Phe-CHO (**1**) and its self-masked aldehyde analogue **2** (Li et al., 2021). Dipeptide aldehyde **1** binds to free enzyme **E** to form an **EI** complex, which apparently undergoes a reaction with the active-site to form a thiohemiacetal adduct (*k*_3_), the EI* complex, which can slowly revert via the *k*_4_ step to re-form the aldehyde. The self-masked aldehyde (**2**), is a *o*-tyrosinyl analogue of **1**, which exists in the solution as a δ-lactol; the form which binds to the protease. The cysteine protease apparently catalyzes the ring-opening of the lactol (The *k*_3_ step), to afford the bound aldehyde (**EI’**), which proceeds to form the reversibly-covalent EI* complex (Li et al., 2021). **(B)** Kinetic scheme for time-dependent inhibitor **I** which competes with substrate **A** for free enzyme **E** to rapidly form complex **EI** (described by inhibition constant *Ki*, orange), which after minutes forms slowly-reversible complex EI* (described by inhibition constant Ki*, blue), for which ***Ki*** > Ki*.

While peptidic aldehydes form reversible thiohemiacetal adducts with the active-site cysteines of cysteine proteases (Cianni et al., 2021; Lewis and Wolfenden, 1977; Pehere et al., 2019; and Woo et al., 1995), and thereby afford exceptionally potent inhibition, free aldehydes are in general too reactive to provide therapeutic agents (Kaplowitz, 2005; Gampe and Verma, 2020; and O'Brien et al., 2005). However, CAT811, a macrocyclic aldehyde which is an inhibitor of the cysteine protease calpain, may be applied topically to the eye leading to the reduction of cataract formation (Morton et al., 2013). Consequently, we developed a novel class of reversible covalent inhibitors of cruzain, known as self-masked aldehyde inhibitors (SMAIs). SMAIs exist in aqueous solutions as stable δ-lactols, and in our hands, cruzain catalyzed the opening of the lactol to provide the enzyme-bound aldehyde, which subsequently formed a thiohemiacetal adduct with cruzain (Figure 1) (Li et al., 2021). These SMAIs provided potent inhibition of cruzain (*K*_i_ = 18–350 nM), and some of these compounds demonstrated potent anti-trypanosomal activity in cellular-infection models of Chagas disease (Li et al., 2021). Given that human cathepsin L and cruzain share 36% amino-acid identity, and consequently, similar peptide-substrate specificity, we sought to characterize these SMAIs as inhibitors of human cathepsin L, and then evaluate potent inhibitors as potential anti-SARS-CoV-2 agents in models of cellular infection. We describe the results of these studies herein. In the preparation of this article, we became aware that benzyl δ-lactols of structures similar to our SMAIs are found in the anti-bacterial, nature-products known as cordycepamides (Fan et al., 2020).

## Materials and Methods

### Chemicals

Sodium acetate, disodium-EDTA, Tris-HCl, and NaCl were obtained from Millipore Sigma. CHAPS and dithiothreitol were obtained from BioGold. Cbz-Phe-Arg-7-amino-4-methylcoumarin (Z-FR-AMC) was purchased from EMD Millipore or GenScript. The methods for the synthesis and characterization of the FRET-based substrate for 3CL-PR, (Abz)HN-Ser-Ala-Val-Leu-Gln*Ser-Gly-Phe-Arg-Lys (ε-Dnp)-CONH_2_ is described in Mellott *et al.* (2021). The synthesis and characterization of all self-masked aldehyde inhibitors described in this work may be found in Li *et al.* (2021).

### Cathepsins

Recombinant proteases were obtained from the following vendors: recombinant human cathepsin L (Millipore Sigma, Athens Research and Technology, Inc.), and human liver cathepsin B (Millipore Sigma), which were used without further purification. The solid proteins were dissolved into a solution of 50 mM sodium acetate (pH 5.5), 1 mM Na_2_EDTA, 1 mM CHAPS, 10% (v/v) DMSO, and 5 mM DTT to final protein concentrations of 1–10 μM in 20-μL aliquots, and stored at −80°C until needed. These samples were then diluted into the same buffer to concentrations of ∼100 nM, and these dilutions were stored at 4°C and used daily until depletion.

### Expression and Purification of SARS-CoV-2 3CL Protease (3CL-PR)

The expression and purification of the 3CL-PR have been previously described (Mellott et al., 2021). In brief, an expression construct of SARS-CoV-2 3CL-PR contained a GST domain at the N-terminus of the 3CL-PR coding sequence, followed by the 3CL-PR cleavage sequence (SAVLQ*SGF) preceding the sequence encoding the remaining 303 amino acids of the 3CL-PR monomer, followed at its C-terminus by a modified PreScission protease sequence (SGVTFQ*GP), that preceded a His_6_ sequence (Zhang et al., 2020; Mellott et al., 2021). Upon expression, auto-proteolysis from 3CL-PR removed the N-terminal GST tag, yielding the authentic N-terminus (SGF). After binding this processed protein to a nickel-NTA column, eluted fractions were pooled and dialyzed to remove imidazole. Proteolysis of the C-terminal H_6_ tag was conducted by incubating with 3.5 units of HRV 3C Protease (Thermo Fisher Scientific) per mg of 3CL-PR (determined by Nanodrop) at 4°C overnight. Subsequently, the protein mixture was subjected to chromatography on a 5-ml GSTrap HP column, and then a 5-ml HisTrap HP column (GE Healthcare), to remove, respectively, the GST-fused HRV 3C protease and undigested H_6_-tagged protein. After chromatography on an anion exchange column and gel-filtration column, the tag-free 3CL-PR was pooled and concentrated (10 kDa molecular weight cutoff filter, GE Healthcare). The protein was deemed to be ≥95% pure by SDS–PAGE, and was stored at –80°C in 12 mM Tris-HCl, 120 mM NaCl, 0.1 mM EDTA, and 2 mM DTT, (pH 7.5) with 50% glycerol (v/v). Analytical gel filtration indicated that native 3CL-PR was the expected homodimer (68 kDa).

### Kinetic Assays and Characterization of Inhibition for Human Cathepsin B and L

For assays of cathepsin L and B, inhibitors were evaluated in 0.25-ml reaction mixtures containing a buffer of sodium acetate (pH 5.5), 1 mM CHAPS, 1 mM Na_2_EDTA, and 5 mM DTT at 25°C. The fluorogenic substrate Cbz-Phe-Arg-7-amino-4-methyl-coumarin (Z-FR-AMC) was dissolved in 100% DMSO, as were all inhibitors and aliquots of both substrates, and inhibitors were added to the reaction mixtures with final concentrations of DMSO of 10% (v/v). Reactions were initiated by addition of the proteases to final concentrations of 1–2 nM of either cathepsin L or cathepsin B. Michaelis constants for Z-FR-AMC were determined for cathepsin L (*K*_m_ = 2.9 μM) and cathepsin B (*K*_m_ = 150 μM), and fixed concentrations of Z-FR-AMC of 1x or 2x*K*_m_ were used to evaluate inhibitors. Formation of the fluorescent product AMC was monitored over 30–60-min time courses for reaction mixtures in 96-well black microplates (Greiner). Rates of peptidolysis of the dipeptide-AMC substrate(s) were measured on either a SpectraMax M2 (Molecular Devices) or a Synergy Mx (Biotek, Wisnooki, VT) microplate reader which measured the formation of fluorescence using an excitation wavelength of λ_ex_ = 360 nm, with detection of emission at λ_em_ = 460 nm at ≥8-s intervals. Control samples excluded substrate. The measured relative fluorescence units (RFUs) of generated AMC were converted to reaction rates of μM/s by use of a standard curve of known AMC concentrations obtained for both plate readers.

### Kinetic Analysis of SARS-CoV-2 3CL-PR and Characterization of Its Inhibitors

In reaction mixtures containing 20 mM Tris-HCl (pH 7.5), 150 mM NaCl, 0.1 mM EDTA, 2 mM DTT, 10% (v/v) DMSO (a final concentration arising from addition of substrates and inhibitors added from 100% (v/v) DMSO solutions), and variable concentrations (10–175 μM) of the FRET-based substrate Abz-SAVLQ*SGFRK (DNP)-NH_2_,^5^ the reaction was initiated by the addition of 3CL-PR to final concentrations of 25–50 nM in 96-well plates (Greiner, flat-bottom half volume, clear black plates). Rates of peptidolysis of the Abz-SAVLQ*SGFRK (DNP)-NH_2_ substrate were measured on either a SpectraMax M5 (Molecular Devices) or a Synergy HTX (Biotek, Wisnooki, VT) microplate reader with λ_ex_ = 320 nm, λ_em_ = 420 nm in 8–60 s intervals, and time courses of inhibition were obtained for either 30 or 60 min intervals. Control samples excluded the substrate. The measured relative fluorescence units (RFUs) of the generated Abz-SAVLQ-COOH were converted to reaction rates of μM/s by use of a standard curve of known concentration of fully hydrolyzed substrates obtained for both plate readers.

### Cell Culture and Evaluation of Efficacy in SARS-CoV-2 Infection

Vero E6 cells [CRL:1586, ATCC], derived from African green monkey cells were grown in an Eagle’s minimal essential medium (EMEM) supplemented with standard doses of penicillin and streptomycin, and 10% fetal bovine serum (FBS), which we designate as the M-10 medium. Human A549 cells that had been stably transduced with human ACE2 viral receptor (A549/ACE2), and then selected for increased ACE2 receptor, were grown in M-10. SARS-CoV-2 (USA_WA1/2020 isolate), the 3rd passage in Vero E6 cells from the original CDC (Atlanta) material with a confirmed sequence, was used throughout the study. A modified Vero E6-based standard micro-neutralization assay was used to rapidly evaluate the drug efficacy against the SARS-CoV-2 infection. Briefly, confluent Vero E6 or A549/ACE2 cells grown in 96-wells microtiter plates were pre-treated with 78 nM to 20 µM of the SMAIs and **K777** (2-fold serially diluted) for 2 h, before infection with ∼100 or ∼500 infectious SARS-CoV-2 particles, respectively, in 100 µL EMEM supplemented with 2% FBS (2-MEM). Cells pre-treated with 2-fold serially-diluted dimethyl sulfoxide (DMSO; final concentration = 1% (v/v)) with or without the virus were included as positive and negative controls, respectively. After cultivation at 37°C for 3 days (Vero E6) or 4 days (A549/ACE2), individual wells were observed by microscopy for the status of a virus-induced formation of the cytopathic effect (CPE). The efficacy of individual drugs was calculated and expressed as the lowest concentration capable of completely preventing virus-induced CPE in 100% of the wells. The values of EC_50_ (the concentration of inhibitor that results in a 50% growth of the virus) were determined in two ways: 1) In duplicate samples of a single compound dilution in which 100% CPE was observed for both replicates, and in which no CPE was observed at the next highest concentration of the inhibitor in duplicates, we assigned the value of EC_50_ as the average of these two concentrations. 2) In cases in which duplicate concentrations result in one sample displaying CPE while the other does not, the value of EC_50_ was assigned to this concentration. All experiments using infectious viruses were conducted at the University of Texas Medical Branch under BSL-3 conditions.

### Fitting of Kinetic Data

The time-course data for compounds which exhibited time-dependent inhibition were fitted to Eq. 1, for which *P* is the relative fluorescence units of the AMC product, where *v*_i_ and *v*_*s*_ are the initial (t < 500 s) and steady-state (t > 3,000 s) velocities, respectively, and *k*_obs_ is the rate constant of conversion of *v*_i_ to *v*_s_, t is time in seconds, and C is a background constant.

$$P=v_{s}t+\left(\frac{v_{i}-\;v_{s}}{k_{obs}}\right)\left(1-e^{-k_{obs}t}\right)+C$$ (1)

Resulting values of *k*_*obs*_ vs. [I] were re-plotted and fitted to Eq. 2, in which *k*_*3*_ and *k*_*4*_ represent the respective rates of formation and reversion of the EI* complex as shown in Figure 1B, in which *K*_*i*_ = *k*_2_/*k*_1_ and *K*_i_* = (*k*_2_/*k*_1_) (*k*_4_/(*k*_3_ + *k*_4_)), and for which A is the fixed substrate concentration, and *K*_a_ is the Michaelis constant.

$$k_{obs}=k_{4}+\frac{k_{3}\left[I\right]}{K_{i}\left(1+\frac{\left[A\right]}{K_{a}}\right)+\left[I\right]}$$ (2)

Linearity of *k*_*obs*_ vs. [I] of the inhibitor compounds will be observed when *K*_*i*_ >> *K*_i_*. Under these conditions, the concentration of inhibitors required to observe time-dependent inhibition would be much less than the value of *K*_*i*_ for the **EI** complex. In this case, Eq. 2 reduces to Eq. 3, which is a linear function with a slope = *k*_*3*_/[*K*_*i*_ (1 + [A]/*K*_a_)] and a *y*-intercept = *k*_4._

$$k_{obs}=k_{4}+\frac{k_{3}\left[I\right]}{K_{i}\left(1+\frac{\left[A\right]}{K_{a}}\right)}$$ (3)

Inhibition constants (*K*_*i*_ and *K*_i_* values) were also obtained by fitting plots of *v*_*i*_/*v*_*0*_ and *v*_*s*_/*v*_*0*_ vs. [inhibitor] to Eqs 4 and 5, wherein *v*_*i*_ and *v*_*s*_ are velocities at, the early and late stages of the time courses of inhibition, respectively, *v*_*0*_ is *v*_*i*_ and *v*_*s*_ when no inhibitor is present, [I] is variable concentrations of the inhibitor, and *K*_*a*_ is the Michaelis constant of the substrate.

$$\frac{v_{i}}{v_{0}}=\frac{1}{\left\{1+\frac{\left[I\right]}{\left[K_{i}\left(1+\frac{\left[A\right]}{K_{a}}\right)\right]}\right\}}$$ (4)

$$\frac{v_{s}}{v_{0}}=\frac{1}{\left\{1+\frac{\left[I\right]}{\left[K_{i}^{*}\left(1+\frac{\left[A\right]}{K_{a}}\right)\right]}\right\}}$$ (5)

## Results and Discussion

### Inhibition of Cysteine Proteases

**FIGURE 2:**
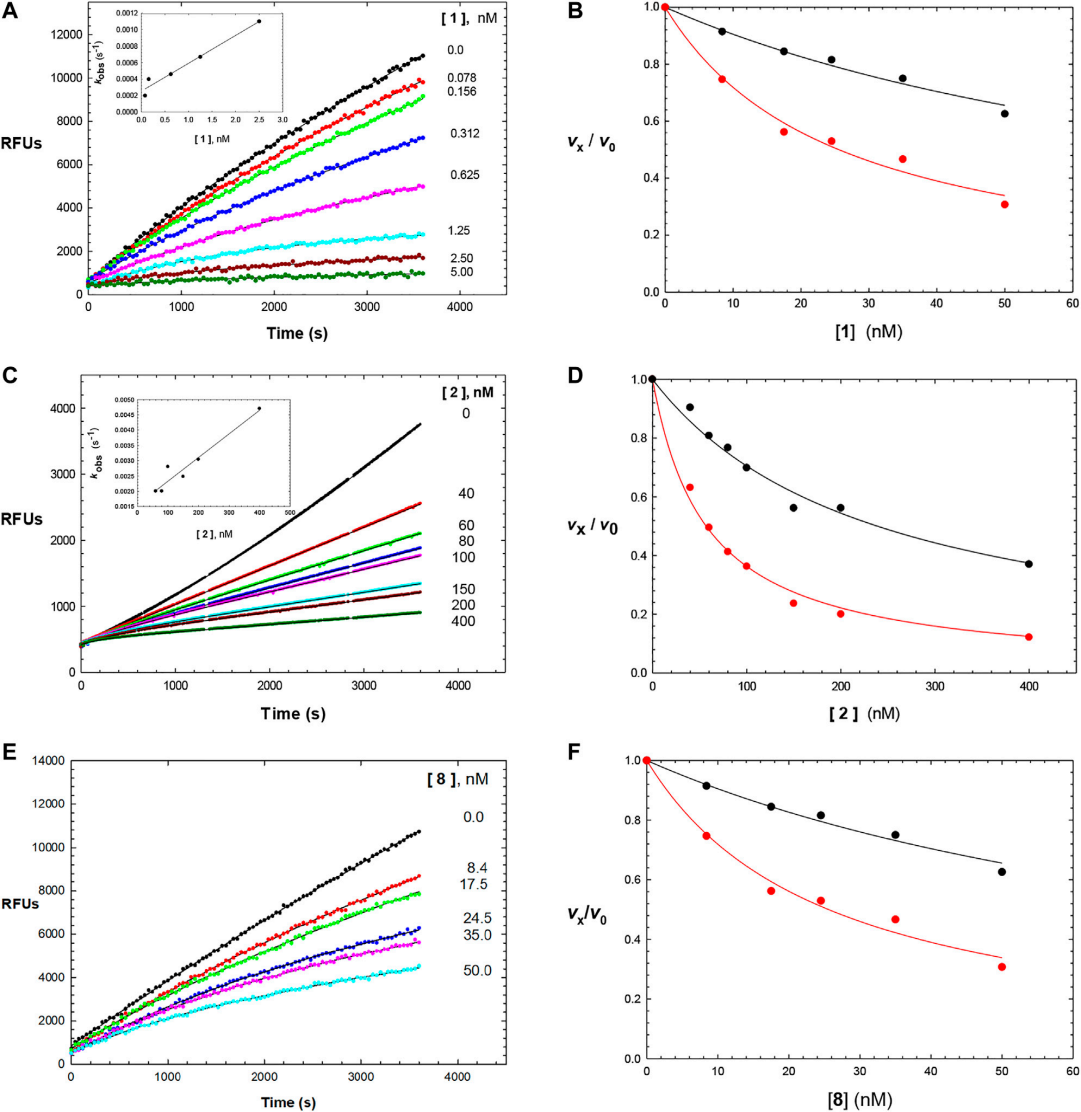
Time courses (0–3600 s) of the inhibition of human cathepsin L by Cbz-Phe-Phe-CHO (**1**) (**(A)**; 0-5 nM of compound **1**), Cbz-Phe-*o*-Tyr-CHO (**2**) (**(C)**; 0–400 nM of compound **2**), and NMe-Pip-Phe-*o*-Tyr-CHO (**8**) (**(E)**; 0–50 nM of compound **8**). The measured RFUs (relative fluorescence units) were obtained upon cleavage of the fluorogenic peptide substrate Cbz-Phe-Arg-AMC (10 μM; **3X** its apparent Michaelis constant with human cathepsin L). The lines drawn through the experimental points were from the plotting of each line to Eq. 1, and resulting values of *v*_i_ and *v*_s_ were normalized by dividing by *v*_i_ and *v*_s_ at [I] = 0 (*v*_0_). to provide values of *v*_i_ /*v*_0_ and *v*_s_/*v*_0_, which were then replotted vs. each concentration of inhibitor as *v*_i_ /*v*_0_ vs. [inhibitor] (black) and *v*_s_/*v*_0_ vs. [inhibitor] (red) in **(B**,**D**,**F)**. Each plot was fitted to versions of the Cheng–Prusoff equation (Eq. 4 for *v*_i_ /*v*_0_ , and Eq. 5 for *v*_s_ /*v*_0_ ), to provide, the apparent inhibition constants of *K*_i_ and *K*_i_*, respectively. The insets in Figures 2A,C are replots of *k*_obs_ vs. [**1**] and *k*_obs_ vs. [**2**], from values of *k*_obs_ obtained from each time-course curve for each inhibitor from fitting to Eq. 1, respectively. The line drawn through the inset plots were from fitting to Eq. 3, resulting in values of *k*_*3*_/[*K*_*i*_ (1 + [A]/*K*_a_)] = (1.5 ± 0.2) × 10^−4^ nM^−1^ s^−1^ and *k*_4_ = (2.6 ± 0.5) × 10^−4^ s^−1^ (inhibitor **1**), and *k*_3_/*K*_i_ = (4 ± 0.5) × 10^−6^ nM^−1^ s^−1^ and *k*_4_ = 0.0016 ± 0.0002 s^−1^ (inhibitor **2**). Fitting of *v*_i_ /*v*_0_ and *v*_s_/*v*_0_ vs. [**8**] resulted in values of *K*_i_ = and *K*_i_* which are found in Table 1, along with all inhibition parameters.

The time courses of the inhibition of human cathepsin L by the parent aldehyde inhibitor in this study, Cbz-Phe-Phe-CHO (**1**), demonstrated initial inhibition, arising from the immediate formation of an **EI** complex, that apparently isomerized to a tighter complex (EI*) in a concentration-dependent manner, after 1,000 s of incubation (Figures 1, 2A), in a manner nearly indistinguishable from that observed for cruzain (Li et al., 2021). By fitting the time course at each concentration of **1** to Eq. 1, we obtained values of *k*_obs_, *v*_i_, and *v*_s_; the latter two of which were normalized by the division of each value by the corresponding value of *v*_i_ and *v*_s_ in which [**1**] = 0, to provide values of *v*_i_/*v*_0_ and *v*_s_/*v*_0_. A replot of the values of *k*_*obs*_ vs. [**1**] (Figure 2A, inset) were fitted to both Eqs 2, 3. The apparent lack of a hyperbolic response of *k*_obs_ by **1** indicated that *K*_i_ was much greater than the applied concentrations of the aldehyde, and that Eq. 3 was the more appropriate equation for data fitting. Consequently, the use of Eq. 3 resulted in values of *k*_*3*_/[*K*_*i*_ (1 + [A]/*K*_a_)] = (1.5 ± 0.2) x 10^−4^ nM^−1^ s^−1^ and *k*_4_ = (2.6 ± 0.5) x 10^−4^ s^−1^, from which we may calculate *k*_4_ *K*_i_/*k*_3_ ∼ *K*_i_* = 13 nM. The rate constant for the reversal of the EI* complex to **EI** is exceptionally slow (*k*_4_ = (2.6 ± 0.5) x 10^−4^ s^−1^), and this is an important factor in the observed, sub-nanomolar value of *K*_i_*, and also reflects the apparent stability of the enzyme-thiohemiacetal complex of EI*. Fitting of the plots of *v*_*i*_/*v*_0_ and *v*_*s*_/*v*_0_ vs. [**1**], Eqs. 4 and 5, resulted in respective inhibition constants of *K*_i_ = 0.18 ± 0.22 nM and *K*_i_* = 0.14 ± 0.01 nM (Figure 2B), respectively, demonstrating that Cbz-Phe-Phe-CHO is an exceptionally potent inhibitor of human cathepsin L (Table 1 and Figure 2A), and is 3-fold more potent than its inhibition of cruzain (*K*_i_* = 0.44 ± 0.04 nM (Li et al., 2021), Table 1).

**TABLE 1:** Self-masked aldehyde inhibitors of cysteine proteases and their effects on SARS-CoV-2 infected cells^a^.

	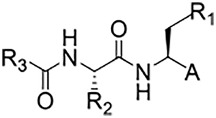				K_i_* (nM)				Anti-CoV-2 EC_50_ (µM)	
Compound	R_4_	R_3_	R_2_	R_1_-A	hCatL	Cruzain^b^	hCatB	3CLpro^b^	Vero E6 Cells	A549/ACE2 Cells
1	H	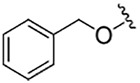	Bz	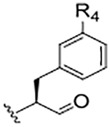	0.14 ± 0.01	0.44 ± 0.02	ND	>10,000	0.47/7.5	>20
2	H	Cbz	Bz	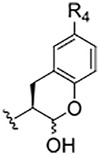	12.6 ± 0.4	49 ± 2	4,500 ± 100	>10,000	10 ± 5	>20
3	OMe	Cbz	Bz	o-Tyr-CHO	17 ± 0.9	350 ± 30	5,500 ± 400	>10,000	ND	ND
4	Me	Cbz	Bz	o-Tyr-CHO	26 ± 0.9	103 ± 5	6,100 ± 900	>10,000	ND	ND
5	CI	Cbz	Bz	o-Tyr-CHO	12.1 ± 0.3	70 ± 10	1400 ± 100	>10,000	ND	ND
6	F	Cbz	Bz	o-Tyr-CHO	10.3 ± 0.5	48 ± 2	2,300 ± 200	>10,000	4	ND
7	CO_2_Me	Cbz	Bz	o-Tyr-CHO	4.8 ± 0.2	18.0 ± 0.5	670 ± 30	>10,000	2.5	ND
8	H	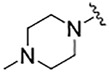	Bz	o-Tyr-CHO	9.0 ± 0.4	47 ± 2	1270 ± 70	>10,000	9 ± 2	ND
9	H	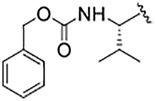	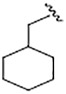	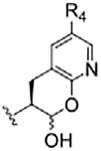	22 ± 4	ND	ND	9 ± 2^b^	ND	3.75
10	H	Me-Pip	Bz	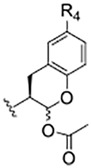	NA	NA	NA	NA	2.5	0.31
11	H	Me-Pip	Bz	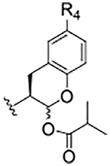	NA	NA	NA	NA	2.5	0.62
12	H	Me-Pip	Bz	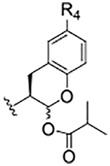	NA	NA	NA	NA	4	0.31
K777^c^	NA	Me-Pip	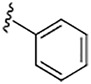	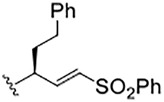	k_inact_/K_I_ = 3 ± 1 µM^−1^-s^−1^	k_inact_/K_I_ = 1.0 ± 0.3 µM^−1^-s^−1^	k_inact_/K_I_ = 0.009 ± 0.004 µM^−1^-s^−1^	>10,000	0.62^c^/0.15	<0.078^c^
PF-07321332			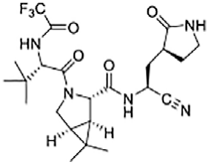		>100mM^d^	ND	>100mM^d^	3.11^d^	0.074^d^/>20	0.077^d^/0.47

a Inhibition constants (K_i_* values) obtained after 30-min incubation of inhibitors with cysteine proteases as described in Methods.

b Data for human cathepsins B and L were obtained at pH 5.5, and at neutral pH for cruzain (pH 7.5) (Zhai and Meek, 2018) and for 3CL protease (pH 7.2).

c Kinetic parameters of inactivation for K777 are as reported in Mellott et al. (2021).

d Data from Owen et al. (2021); NA, not applicable; ND, no data.

Its analogue, the self-masked aldehyde Cbz-Phe-*o*-Tyr-CHO (**2**) demonstrated less time-dependent inhibition compared to **1** (Figure 2C), and again, the replot of *k*_*obs*_ vs. [**2**] (Figure 2C, inset) was linear. Fitting of these data to Eq. (3) resulted in the values of *k*_3_/*K*_i_ = (8 ± 1) x 10^−6^ nM^−1^ s^−1^ and *k*_4_ = 0.0016 ± 0.0002 s^−1^, leading to *k*_4_ *K*_i_/*k*_3_ ∼ *K*_i_* = 200 nM. The conversion of the apparent EI* to EI (rate constant of *k*_4_ = 0.0016 ± 0.0002 s^−1^) is ∼5-fold faster than that of aldehyde **1**, which contributes to the 100-fold weaker inhibition of **2**, as exemplified by *K*_i_^*^ (see below). As discussed in our study of compound **2** with cruzain, we ascribed this faster rate of reversion of the thiohemiacetal (EI* to EI) due to the facilitation of the phenoxide group of **2** to break the C–S bond of the thiohemiacetal to elicit re-formation of the lactol of **2** (Li et al., 2021). Fitting of plots of *v*_*i*_/*v*_0_ and *v*_*s*_/*v*_0_ vs. [**2**] to, Eqs 4 and 5, resulted in respective inhibition constants of *K*_i_ = 54 ± 6 nM and *K*_i_* = 12.6 ± 0.4 nM. Also, as with cruzain, the self-masked aldehyde analogue of **1** (Cbz-Phe-*o*-Tyr-CHO (**2**)) was 100-fold less potent as an inhibitor of cathepsin L, but the value of *K*_i_* of **2** for cathepsin L was 3.9-fold more potent than that observed for cruzain (Table 1). The kinetic behavior of the inhibitors of **1** and **2** are very similar to that observed with cruzain (Li et al., 2021).

Addition of the electron-donating groups methoxy (**3**) and methyl (**4)** at the 4′-position of the *o*-Tyr group resulted in respective 1.4-fold and 2-fold increases in their *K*_i_* values compared to **2**, while the substitution of **2** at this position with the electron-withdrawing groups fluoro (**5**) and chloro (**6**) had little effect on the inhibition of cathepsin L compared to unsubstituted compound **2**. With the exception of compound **3,** these inhibitors were ≥2-fold more potent for human cathepsin L than cruzain, which also indicated that substitution at the 4′-position had less of an effect on the inhibition of cathepsin L than that of cruzain. Like with cruzain, the 4′-methylcarboxylate substituent of compound **7** improved potency 2.6-fold over that of **2**, and afforded the most potent SMAI in this series of compounds. Substitution of the terminal Cbz group of **2** with an N-methyl-piperidinyl urea provided a more potent analogue of **2**: compound **8** (Figures 2E and F), for which values of *K*_i_ = 22 ± 1 nM and *K*_i_* = 9.0 ± 0.4 nM were obtained. Inhibitor **8** has greater aqueous solubility than **2** (Li et al., 2021), and was nearly twice as potent as **2** for human cathepsin L. In the earlier study, we showed that the [*aldehydic*-^13^C] form of compound **8**, as analyzed by ^1^H -^13^C HSQC NMR, remained exclusively in its δ-lactol form in aqueous reaction buffer, but upon the addition of an equimolar amount of cruzain, changes to the chemical shifts in the NMR data were consistent with the formation of an enzyme-bound thiohemiacetal adduct (Li et al., 2021). The improved potency of compound **8** with cathepsin L suggests, but does not prove, that cathepsin L in kind, catalyzes the opening of the δ-lactol form leading to the formation of a thiohemiacetal with cathepsin L.

Where studied, the SMAIs in Table 1 were 120–320-fold less potent inhibitors of human cathepsin B than with cathepsin L. This selectivity also underscores the 36% amino-acid identity between cruzain and human cathepsin L, while there is only a 29% amino-acid identity between human cathepsins L and B. Expectantly, none of compounds **1**–**8** exhibited inhibition of the SARS-CoV-2 3CL protease at concentrations of 10 μM or lower (Table 1).

### Modifications of SMAIs to Develop 3CL-PR Inhibitors That are Also Inhibitors of Human Cathepsin L

We sought to identify an amino-acid sidechain at the P_1_ position of a peptide scaffold which would be recognized by both human cathepsin L and SARS-CoV-2 3CL PR. To date, only 2-oxopyrrolidinyl-alanyl groups (Op-Ala), in effect, glutaminyl lactams, have afforded suitable sidechains at the P_1_ position for inhibitors of SARS-CoV-2 3CL-PR (Dai et al., 20202020; Zhang et al., 2020; and Owen et al., 2021). Given that the substituted or un-substituted phenylalanyl groups at the P_1_ position within the peptidomimetic scaffolds of compounds **1**–**8** provide potent cathepsin L inhibitors that had no effect on purified 3CL protease, we sought to find an amino-acid group for the P_1_ sidechain that would be accommodated by both human cathepsin L and SARS-CoV-2 3CL protease. Such bi-functional anti-CoV-2 agents would presage a novel class of COVID-19 drugs that might subvert the development of viral mutations that thwart the action of drugs that only target 3CL protease, or for that matter, cathepsin L.

We hypothesized that compound **9** would be an inhibitor of both 3CL protease and human cathepsin L. In this SMAI, the P_3_ position contains a valyl group, the P_2_ position contains the leucyl analogue cyclohexyl-alanyl, and the P_1_ position contains the novel glutamine analogue, 2-pyridone (Li et al., 2021). We expected the 2-pyridone aldehyde to also form a δ-lactol, and also be recognized by both human cathepsin L and SARS-CoV-2 3CL protease, and this was indeed the case. Compound **9** is a low nanomolar inhibitor of both enzymes (*K*_i_ = 22 nM for human cathepsin L, and *K*_i_ = 9 nM for SARS-CoV-2 3CL protease), demonstrating the feasibility of designing an inhibitor to target both enzymes.

### Prodrugs of SMAI 8

**SCHEME 2:**
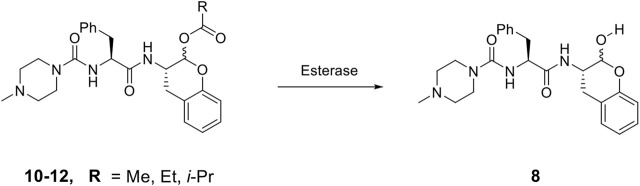
De-acylation of Pro-drugs of Compound 8 as catalyzed by cellular esterases.

As compound **8**, an inhibitor with a peptidomimetic scaffold similar to that of the anti-chagasic compound **K777** (common elements include the P_3_ N-methyl-piperazinoyl group and the P_2_ phenylalanyl group), demonstrated potent inhibition of human cathepsin L, we sought to protect the δ-lactol functionality of **8** by synthesizing pro-drug forms in which the secondary alcohol of **8** was acylated. (Li et al., 2021) (Scheme 2). Compounds **10**–**12**, respectively comprise the O-acetyl, O-*n*-propanoyl, and the O-iso-butanoyl derivatives of lactol **8**. We previously demonstrated that treatment of pro-drugs **10–12** with porcine esterase *in vitro* generated inhibitor **8** (Scheme 1). Pro-drugs **10–12** exerted anti-trypanosomal activity at micromolar concentrations in infected-cell cultures which was superior to that of the free lactol **8** (Li et al., 2021). From these results we sought to evaluate these pro-drug forms of **8** in SARS-CoV-2-infected mammalian cells, as described below.

### Anti-SARS-CoV-2 Activities of the SMAIs

**FIGURE 3:**
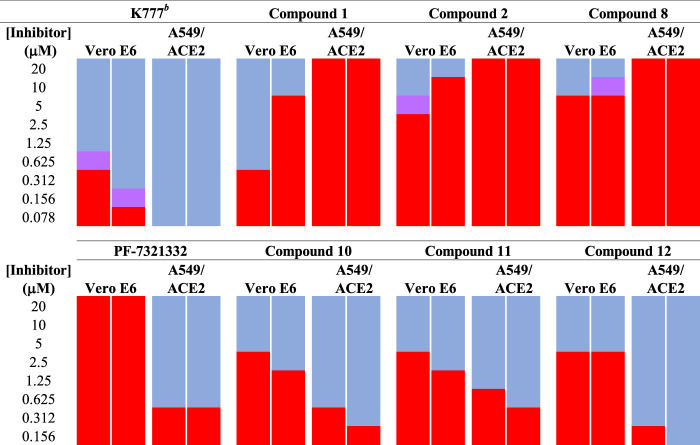
Anti-SARS-CoV-2 activity of SMAIs, compared to that of **K777** and **PF-07321332**. SARS-CoV-2-infected Vero E6 and human A549/ACE2 cells were treated as described with **K777**, aldehyde **1**, SMAIs **2**, **8**, and the pro-drug forms of **8**, compounds **10-12**.

We tested aldehyde **1**, selected SMAIs (compounds **2, 8, 10–12**), and the approved drug **PF-07321332**, in two types of mammalian cells (Vero E6 and A549/ACE2) infected with SARS-CoV-2 (Figure 3 and Table 1), along with data for **K777** as previously reported (Mellott et al., 2021). Vero E6 are derived from African green monkeys, while A549/ACE2 cells are human adenocarcinoma cells that are stably transduced with the human ACE2 viral receptor. We previously found that **K777,** an irreversible inactivator of human cathepsin L blocked the SARS-CoV-2-induced cytopathic effect (CPE) in Vero E6 and A549/ACE2 cells at respective values of EC_50_ = 625 and <78 nM. In a second study, an EC_50_ of 156 nM was obtained in Vero E6 cells. These activities of **K777** are among the most potent yet observed for any inhibitor of SARS-CoV-2 cell infection. The anti-SARS-CoV-2 effects of **PF-07321332** in infected Vero and A549/ACE2 cells were reported as respective EC_50_ values of 74 and 77 nM. However, in our hands, **PF-07321332** had no anti-SARS-CoV-2 effects in Vero E6 cells at concentrations of ≤20 μM, while we measured a value of EC_50_ = 470 nM in A549/ACE2 cells, which is >6-fold less potent than **K777**. Regardless of the reasons for these differences obtained in two different laboratories, we consider the results we report in our studies to serve as “benchmark” data for an approved drug to compare with our inhibitors.

Dipeptide aldehyde **1** was a potent anti-SARS-CoV-2 agent in Vero E6 cells (EC_50_ = 470 nM), but upon repeating this experiment with a different sample of **1**, we observed a value of EC_50_ = 7.5 μM. The cause of this discrepancy is unknown, as the experimental conditions are identical. Compound **1** exerted no inhibition of CPE in A549/ACE2 at concentrations of ≤20 μM, which may validate the higher of the two EC_50_ values observed in Vero E6 cells. The value of EC_50_ for SMAI **2** was found to be 10 ± 5 μM in Vero E6 cells, while in A549/ACE2 cells, there was no inhibition of CPE at concentrations of ≤20 μM. The 4′-benzyl-substitituted SMAIs **6** and **7** displayed a slightly higher anti-CoV-2 activity in Vero E6 cells, with respective values of EC_50_ = 4 and 2.5 μM in infected Vero E6 cells (Table 1). The more soluble SMAI, compound **8**, had a slightly increased anti-SARS-CoV-2 activity compared to SMAI **2** in Vero E6 (EC_50_ = 9 ± 2 μM), but again, no anti-viral effect in infected A549/ACE2 cells. We previously reported (Li et al., 2021) that the dual inhibitor of human cathepsin L and 3CL-PR, compound **9**, had no effect in SARS-CoV-2-infected Vero E6 cells, but demonstrated an EC_50_ = 3.75 μM in SARS-CoV-2-infected A549/ACE2 cells (Table 1).

Two of the three pro-drug analogues of compound **8**, compounds (**10**–**12**) proved the most potent anti-SARS-CoV-2 agents in our study. These inhibitors differ structurally from compound **8** only in that the secondary alcohol of the lactol of **8** is now O-acylated with, acetyl, propanoyl, and iso-butanoyl groups, respectively. As previously proposed (Li et al., 2021), we expected that O-acylation of this hydroxyl group would provide protection of the lactol from opening to the requisite aldehyde, and subsequent reaction with electrophiles within cells. Indeed, pro-drugs **10**–**12** were highly effective in preventing CPE in both Vero E6 (respective EC_50_ values of 2.5, 2.5, and 3.75 μM) and especially in A549/ACE2 cells (respective EC_50_ values of 312, 625, and 156 nM). It is notable that in A549/ACE2 cells, the more sterically-hindered O-iso-butanoyl group provided the most potent inhibition of CPE, suggesting that this ester has the highest metabolic stability in cells of the three pro-drugs studied. It is noteworthy that these three pro-drugs of SMAI **8** are effectively equipotent with that of **PF-07321332** in A549/ACE2 cells, and demonstrated an activity at low micromolar concentrations in CoV-2-infected Vero E6 cells, in which **PF-07321332** was inactive.

## Summary

In this report we have described the expanded use of our novel class of cysteine protease inhibitors, the self-masked aldehydes, to the inhibition of human cathepsin L, in which these inhibitors were more potent than their inhibition of the highly similar cysteine protease, cruzain, from the parasitic protozoan *Trypanosoma cruzi*. While none of the SMAIs inhibited the 3CL protease of SARS-CoV-2, some nevertheless demonstrated inhibition of the cytopathic effect arising from SARS-CoV-2 infection of two mammalian cell lines. SMAI **8** exhibited a value of *K*_i_ = 9 nM vs. human cathepsin L, but with moderate anti-SARS-CoV-2 activity at micromolar concentrations in Vero E6 cells, and with no effect in the SARS-CoV-2-infected A549/ACE2 cells. However, three O-acylated pro-drug forms of **8** blocked CoV-2-mediated CPE in infected A549/ACE2 cells with potencies equivalent to that of the FDA-approved, 3CL-PR inhibitor **PF-07321332**. As we have embarked on the discovery of a single SMAI that inhibits both hCatL and SARS-CoV-2 3CL protease, as exemplified here by the 2-pyridone analogue of alanine compound **9**, which, while a nanomolar inhibitor of both enzymes, the anti-CoV-2 effects of this dual inhibitor are no better than that of SMAIs discussed previously. However, based on the success of pro-drug forms of human cathepsin L inhibitor **8**, it would be circumspect to synthesize O-acylated analogues of SMAI **9**, and evaluate them in cellular models of the SARS-CoV-2 infection. This will be the focus of upcoming studies.

## Data Availability

The raw data supporting the conclusion of this article will be made available by the authors, without undue reservation.

## Abbreviations

AMC, 7-amino-4-methylcoumarin; CHAPS, 3-[3-(cholamidopropyl)dimethylammonio]-1-propanesulfonate; Cbz/Z, benzyloxycarbonyl; DMSO, dimethyl sulfoxide; DTT, dithiothreitol; EDTA, ethylenediaminetetraacetic acid; SMAI, self-masked aldehyde inhibitor; Z-FR-AMC, carboxybenzyl-L-phenylalanine-L-arginine-7-amino-4-methylcoumarin.
